# Equity in HIV testing: evidence from a cross-sectional study in ten Southern African countries

**DOI:** 10.1186/1472-698X-10-23

**Published:** 2010-09-13

**Authors:** Steven Mitchell, Anne Cockcroft, Gilles Lamothe, Neil Andersson

**Affiliations:** 1 CIETcanada, 1 Stewart Street, Ottawa, Ontario, Canada; 2 CIET Trust Botswana, PO Box 1240, Gaborone, Botswana; 3 Department of Mathematics and Statistics, University of Ottawa, Canada; 4 Centro de Investigación de Enfermedades Tropicales, Universidad Autónoma de Guerrero, Acapulco, Mexico

## Abstract

**Background:**

HIV testing with counseling is an integral component of most national HIV and AIDS prevention strategies in southern Africa. Equity in testing implies that people at higher risk for HIV such as women; those who do not use condoms consistently; those with multiple partners; those who have suffered gender based violence; and those who are unable to implement prevention choices (the choice-disabled) are tested and can have access to treatment.

**Methods:**

We conducted a household survey of 24,069 people in nationally stratified random samples of communities in Botswana, Lesotho, Malawi, Mozambique, Namibia, South Africa, Swaziland, Tanzania, Zambia, and Zimbabwe. We asked about testing for HIV in the last 12 months, intention to test, and about HIV risk behaviour, socioeconomic indicators, access to information, and attitudes related to stigma.

**Results:**

Across the ten countries, seven out of every ten people said they planned to have an HIV test but the actual proportion tested in the last 12 months varied from 24% in Mozambique to 64% in Botswana. Generally, people at higher risk of HIV were not more likely to have been tested in the last year than those at lower risk, although women were more likely than men to have been tested in six of the ten countries. In Swaziland, those who experienced partner violence were more likely to test, but in Botswana those who were choice-disabled for condom use were *less *likely to be tested. The two most consistent factors associated with HIV testing across the countries were having heard about HIV/AIDS from a clinic or health centre, and having talked to someone about HIV and AIDS.

**Conclusions:**

HIV testing programmes need to encourage people at higher risk of HIV to get tested, particularly those who do not interact regularly with the health system. Service providers need to recognise that some people are not able to implement HIV preventive actions and may not feel empowered to get themselves tested.

## Background

An equitable health care approach is largely concerned with making services available and accessible to those who need them most in order to reduce unfair and avoidable gaps in access and uptake [1,2]. Typically, this means people living in rural areas, women, children and the poor. But these vulnerable groups are not homogenous, and inequities within them can further compound discrepancies in uptake of services and quality of life [3,4].

There is a generalised AIDS epidemic in many countries of southern Africa and increased testing for HIV is an important adjunct to prevention strategies. Knowledge of one's own HIV status is an individual right that should be independent of socioeconomic status. This knowledge can lead to timely access to antiretroviral therapy (ART) which can lengthen and improve quality of life. There is also evidence that people who know they are HIV positive modify their behaviour, increasing condom use and reducing their number of sexual partners [5]. HIV testing of pregnant women is the first step in accessing prevention of mother to child transmission (PMTCT) services. However, testing can also lead to increased intimate partner violence particularly if the result is positive [6,7]. There is also some evidence of continued or even escalated risky behaviour among those who test negative [8].

HIV testing coupled with counseling is a component of most national HIV and AIDS prevention strategies in southern Africa [9]. For example, Botswana offers both voluntary counseling and testing (VCT) at testing centres and routine "opt-out" HIV testing (RHT) in government health facilities where most patients are routinely tested for HIV after an explanation of the test, but have the right to refuse the test [10]. However, uptake of HIV testing remains low in many African countries. The World Health Organization reported that in 2007 only one in ten people in sub-Saharan Africa had been tested and knew their HIV status [11].

HIV testing is informative for the equity discourse. People in southern Africa are more likely to be tested if they live in urban areas [1,12], if they are less poor [13,14], and if they have more education [15-17]. Those who perceive themselves to be at risk of HIV are reported to be more likely to be tested [18,19], as are those who believe ART can help an HIV positive person live longer [20]. Other authors have reported that HIV and AIDS stigma can be an important barrier to testing [21-23].

To achieve equity in testing and to optimise the role of testing in prevention, strategies should ensure people who are at higher risk for HIV are tested. These include women [24,25], people who do not always use condoms - particularly with non-regular or casual partners [26], people with multiple sexual partners [27,28], and people who have experienced intimate partner violence [25,29]. Some people may be aware of what they need to do to protect themselves but are not able to implement their protection choices: the choice disabled [30]. Such people are also likely to be at higher risk of HIV infection.

A 2007 cross-sectional survey in ten southern African countries provided an opportunity to investigate factors associated with uptake of HIV testing. We examined whether people at higher risk of HIV came forward for testing more than people at less risk of HIV, taking into account other factors related to HIV testing.

## Methods

### Data collection and analysis

In 2007, a household survey covered nationally representative cluster samples in ten Southern African countries: Botswana, Lesotho, Malawi, Mozambique, Namibia, South Africa, Swaziland, Tanzania, Zambia and Zimbabwe. In each country, we selected a stratified last stage random sample of enumeration areas (EA) of the most recent census, stratified by rural, urban, and capital location. Field teams interviewed people aged 16-60 years in about 100 households in each EA, covering all contiguous households radiating from a random starting point. For country specific estimates, we calculated weights to ensure sample proportions reflected those of the national populations in rural, urban and capital locations. We weighted regional estimates to adjust for the differing total populations of the 10 countries.

The administered questionnaire asked about: age, sex, and educational status; condom use with regular and non-regular partners; number of sexual partners; perception of personal HIV risk; HIV testing in the last 12 months, intention to have an HIV test; and experience of intimate partner violence in the last 12 months (see Additional file 1: Questionnaire). After data collection, trained operators entered the data twice with validation using Epi Info [31]. Analysis relied on CIETmap open source software [32].

Analysis focused on HIV testing uptake, defined as having gone for a test in the last 12 months. We examined associations between uptake and potentially related factors in bivariate and then multivariate analysis using the Mantel Haenszel procedure [33], which tests the conditional independence of two dichotomous variables while controlling for confounders using stratification. It is analogous to fixed effects modelling. We adjusted for clustering using a method described by Gilles Lamothe based on a variance estimator to weight the Mantel Haenszel odds ratio for cluster-correlated data. The Mantel Haenszel methods do not properly control for sample-to-sample variability in the presence of clusters which might have a random effect on the variables involved in study [34]. In the sampling and surveys literature, a cluster adjustment of the Mantel Haenszel odds ratio using the delta-method has been proposed in the context of complex sample surveys [35]. This type of cluster adjustment can be used in our context of modeling multinomially clustered data. The cluster adjustment uses the delta-method with the between cluster variance estimate [36,37] to obtain a cluster adjusted interval estimate for the Mantel Haenszel odds ratio.

We considered several categories of people to be at higher risk of HIV: females; those who did not *always *use a condom for sex with a non-regular partner; those who had more than one sexual partner in the last 12 months; those who had experienced intimate partner violence in the last 12 months; and those who were choice-disabled for condom use. We defined people as "condom choice disabled" if they said they would have sex if their partner refused to use a condom *and *(in answer to a separate question) believed their partner could be at risk of HIV, representing those who felt they could not protect themselves despite being aware of the risk of HIV infection. We examined the associations between these aspects of being at higher risk of HIV and being tested for HIV. The analysis also took into account other variables likely to be related to being tested for HIV: age, education, urban or rural residence, household income, food sufficiency as an indicator of poverty (had enough food to eat in the last week), sources of information about HIV and AIDS (such as TV, radio, print materials, family, friends, clinic, church), having discussed HIV or AIDS in the last year, belief that ART can help someone with AIDS live longer, and stigmatising attitudes (belief that HIV is punishment for sinning, and belief that people with HIV should live apart from others).

All reported frequencies are weighted. We performed analysis for each country separately, using the same initial model for all countries. Associations in the final models are described by the adjusted odds ratio (OR) and 99% cluster-adjusted confidence intervals (CIca). A further analysis examined factors related to intention to test among those who had not been tested in the last year, in a single model across the 10 countries.

We imputed 10 additional datasets using the Amelia II program for missing data [38] to test how missing data would effect the final models. These tests showed little effect on the final models, so we report the original results.

### Ethical review

An accredited international ethical review board approved the project as did an ethical review board in each country: the Health Research and Development Committee, Ministry of Health in Botswana (certificate no. PPME-13/18/1 Vol I (148), dated May 30 2007); Research and Ethics Committee, Ministry of Health and Social Welfare in Lesotho (certificate dated July 09 2007); the National Health Sciences Research Committee, Ministry of Health in Malawi (certificate no. MED/4/36c, dated 16 August2007); the Comite Nacional de Bioetica para a Saude, Ministerio da Saude in Mozambique (certificate no. 177/CNBS/07, dated 31 August 2007); the Research Management Committee, Ministry of Health and Social Services in Namibia (certificate no. 17/3/AP, dated 11 July 2007); the CIETafrica Research Ethics Committee in South Africa (certificate dated 23 April 2007); the Scientific and Ethics Committee, Ministry of Health and Social Welfare in Swaziland (certificate no. MH/599B, dated 19 July 2007); the Institutional Review Board, Ifakara Health Research and Development Centre in Tanzania (certificate no. IHRDC/IRB/No. A006-2007, dated 8 June 2007); the Permanent Secretary, Ministry of Health, Zambia (certificate dated 24 June 2007); and the Medical Research Council of Zimbabwe (certificate no. MRCZ/A/1383, dated 28 June 2007).

## Results

### Evidence base

The field teams approached a total of 37341 households. Some 9118 of these (24%) had no one home, 3119 (8%) had no members present aged 16-60, and 3565 (10%) refused to participate in the survey. Some 21539 households (58% of those originally approached) participated in the survey. Within the households, interviewers identified 57822 people aged 16-60 years from 269 sites across the ten countries. They interviewed 24069 (42%) of these people. Some 4065 (7%) refused to be interviewed, and the remaining 29688 (51%) were absent at the time of the visit. Across the 10 countries, two-thirds (15886/24023) of the respondents were female, half (11529/23852) had primary education or less, and one-quarter (6836/23933) did not have enough food to eat in the last week, with some variation between countries (Table 1).

**Table 1:** Characteristics of survey respondents - weighted % (n)

Country	respondents	Rural residence	female	Primary Education or less	Not enough food in the last week
Botswana	2559	45 (1091/2559)	70 (1784/2552)	30 (755/2524)	33 (846/2551)
Lesotho	2097	71 (1534/2097)	71 (1476/2094)	48 (1001/2089)	32 (693/2088)
Malawi	2358	87 (2016/2358)	70 (1655/2356)	72 (1675/2351)	19 (424/2350)
Mozambique	2627	70 (1590/2627)	55 (1437/2618)	76 (1911/2610)	30 (774/2608)
Namibia	2167	56 (1141/2167)	66 (1426/2165)	28 (594/2148)	23 (482/2149)
South Africa	2283	61 (1409/2204)	69 (1565/2271)	23 (527/2250)	27 (634/2264)
Swaziland	1975	80 (1504/1975)	73 (1433/1970)	37 (710/1956)	40 (762/1960)
Tanzania	2934	80 (2467/2934)	61 (1803/2933)	84 (2482/2916)	17 (557/2920)
Zambia	2578	73 (1725/2578)	65 (1682/2577)	47 (1153/2532)	25 (634/2568)
Zimbabwe	2491	63 (1475/2491)	65 (1625/2487)	29 (721/2476)	41 (1030/2475)
*Region*	*24069*	*62* *(15338/23990)*	*66* *(15886/24023)*	*51* *(11529/23852)*	*25* *(6836/23933)*

### Variables related to HIV risk

Table 2 shows the proportion of respondents from each country who did not always use a condom with a non-regular partner, out of all respondents (including those who did not have a non-regular partner). Nearly a third (29%) of all respondents said they did not always use a condom with a non-regular partner, with great variation between countries, from 7% in Swaziland to 64% in Mozambique. Some 18% of respondents were choice-disabled for condom use, and 15% had more than one sexual partner in the last 12 months (among all respondents, whether they had a partner or not). The proportion reporting multiple partners varied from 6% in Malawi to 25% in Lesotho. At least one in three respondents from each country felt they were at risk of HIV (or were uncertain of their risk). In three countries - Botswana, Mozambique, and Swaziland - more than half the respondents thought they were, or could be, at risk of HIV. Some 15% of respondents reported intimate partner violence in the last 12 months, with little variation between countries..

**Table 2:** HIV risk characteristics among all respondents (including those with no partner) - weighted % (n)

Country	do not always use a condom with a non- regular partner	choice- disabled for condom use*	more than one partner in the last year	Experienced partner violence in the last year	feel they are, or could be, at risk of HIV
Botswana	12 (289/2400)	10 (242/2391)	20 (489/2450)	15 (373/2510)	50 (1244/2491)
Lesotho	26 (532/2047)	37 (647/1807)	25 (493/2027)	16 (322/2067)	47 (950/2059)
Malawi	30 (689/2319)	9 (210/2263)	6 (149/2318)	13 (305/2332)	35 (825/2308)
Mozambique	64 (1587/2475)	36 (829/2387)	20 (498/2461)	15 (384/2522)	53 (1336/2495)
Namibia	15 (331/2141)	14 (289/2046)	17 (371/2123)	14 (294/2146)	46 (1003/2137)
South Africa	23 (458/2014)	11 (210/1991)	16 (322/2049)	14 (306/2149)	33 (710/2140)
Swaziland	7 (137/1852)	18 (321/1752)	10 (185/1839)	14 (268/1913)	52 (984/1891)
Tanzania	25 (720/2919)	22 (573/2731)	17 (502/2914)	17 (473/2921)	32 (906/2884)
Zambia	27 (693/2469)	26 (636/2368)	12 (288/2504)	20 (513/2528)	47 (1205/2515)
Zimbabwe	21 (490/2289)	15 (343/2271)	10 (228/2312)	17 (388/2378)	40 (942/2342)
*Region*	*29* *(5926/22925)*	*18* *(4300/22007)*	*15* *(3525/22997)*	*15* *(3626/23466)*	*37* *(10105/23262)*

*Those who said they would have sex if their partner refused to use a condom and believed their partner to be at risk of HIV

### HIV Testing

Most respondents (90%, 21010/23376) knew where to get an HIV test. Nearly all respondents from Botswana (97%), Malawi (97%) and Namibia (96%) knew where to be tested, and at least 85% knew in the other countries, except in Mozambique, where 70% (1811/2505) knew where to get an HIV test.

Across the 10 countries, seven out of ten people (71%) said they planned to have an HIV test but only 38% had been tested in the last 12 months. The proportion tested in the last 12 months varied from only 24% in Mozambique to 64% in Botswana (Table 3).

**Table 3:** HIV testing and intention to test - weighted % (n)

Country	had an HIV test in the last year	plan to have an HIV test
Botswana	64 (1583/2498)	93 (2313/2503)
Lesotho	33 (645/2055)	78 (1588/2062)
Malawi	46 (1035/2326)	74 (1709/2326)
Mozambique	24 (639/2489)	64 (1618/2501)
Namibia	49 (1057/2142)	84 (1812/2148)
South Africa	46 (993/2132)	66 (1421/2143)
Swaziland	44 (835/1897)	81 (1525/1897)
Tanzania	31 (893/2914)	78 (2248/2920)
Zambia	36 (949/2519)	74 (1915/2523)
Zimbabwe	31 (734/2352)	63 (1473/2349)
*Region*	*38* *(9363/23324)*	*71* *(17622/23372)*

### Factors related to uptake of testing

Table 4 shows the final models of the multivariate analysis for those who had been tested in the last year for each country. Males were significantly less likely than females to have been tested in the last year in all countries except Lesotho, Malawi, South Africa and Tanzania. In Swaziland only, those who had experienced partner violence were significantly more likely to have been tested than those who had not experienced partner violence. In Botswana, those who were choice-disabled for condom use were significantly *less *likely to have been tested than those who were not choice-disabled for condom use. Otherwise, none of the HIV risk factors we examined (multiple partners, lack of condom use, intimate partner violence and condom use choice disablement) were significantly associated with having had an HIV test, taking other factors into account.

**Table 4:** Final multivariate models of variables associated with testing uptake by country* (Adjusted OR and cluster adjusted confidence intervals)

Botswana	Adjusted OR	99%CIca for Adjusted OR
Male sex	0.70	0.56-0.87
Choice-disabled for condom use	0.58	0.34-0.99
Learned about HIV/AIDS from church	1.35	1.04-1.76
Learned about HIV/AIDS from clinic/health centre	2.84	1.68-4.82
Learned about HIV/AIDS from family	1.59	1.16-2.19
Talked often to others about HIV/AIDS	1.46	1.09-1.94

**Lesotho**	**Adjusted OR**	**99%CIca for Adjusted OR**

Learned about HIV/AIDS from print materials	1.49	1.06-2.11
Learned about HIV/AIDS from church	1.46	1.04-2.04
Learned about HIV/AIDS from clinic/health centre	2.06	1.27-3.34
Learned about HIV/AIDS from family	1.48	1.07-2.05
Talked often to others about HIV/AIDS	1.77	1.27-2.46
Feels telling people you are HIV positive helps	1.58	1.21-2.06
Knows that ARVs can help a person live longer	1.88	1.39-2.56

**Malawi**	**Adjusted OR**	**99%CIca for Adjusted OR**

16 - 39 years of age	1.78	1.27-2.49
Learned about HIV/AIDS from print materials	1.87	1.46-2.39
Learned about HIV/AIDS from clinic/health centre	2.25	1.19-4.24
Talked often to others about HIV/AIDS	1.60	1.14-2.25

**Mozambique**	**Adjusted OR**	**99%CIca for Adjusted OR**

16 - 39 years of age	1.67	1.13-2.47
Male sex	0.63	0.50-0.80
Secondary education or above	1.63	1.17-2.27
Learned about HIV/AIDS from television	1.73	1.25-2.40
Learned about HIV/AIDS from print materials	1.42	1.06-1.91
Learned about HIV/AIDS from clinic/heath centre	1.99	1.27-3.12
Talked often to others about HIV/AIDS	1.81	1.35-2.44
Knows that ARVs can help a person live longer	1.78	1.22-2.60

**Namibia**	**Adjusted OR**	**99%CIca for Adjusted OR**

Male sex	0.56	0.45-0.69
Learned about HIV/AIDS from television	1.51	1.18-1.94
Learned about HIV/AIDS from church	1.35	1.06-1.73
Learned about HIV/AIDS from clinic/heath centre	1.77	1.11-2.83
Talked often to others about HIV/AIDS	1.78	1.49-2.14

**South Africa**	**Adjusted OR**	**99%CIca for Adjusted OR**

Learned about HIV/AIDS from clinic/health centre	2.45	1.57-3.82
Learned about HIV/AIDS from family	1.73	1.21-2.48

**Swaziland**	**Adjusted OR**	**99%CIca for Adjusted OR**

Male sex	0.49	0.35-0.70
Learned about HIV/AIDS from clinic/health centre	3.39	2.00-5.72
Talked often to others about HIV/AIDS	1.48	1.16-1.90
Knows that ARVs can help a person live longer	1.91	1.32-2.75
Experienced partner violence	1.61	1.08-2.40

**Tanzania**	**Adjusted OR**	**99%CIca for Adjusted OR**

Secondary education or above	1.29	1.04-1.62
Learned about HIV/AIDS from clinic/health centre	2.00	1.49-2.69
Talked often to others about HIV/AIDS	1.56	1.12-2.15
Would tell someone if they were HIV positive	1.55	1.06-2.28
Knows that ARVs can help a person live longer	1.60	1.15-2.24

**Zambia**	**Adjusted OR**	**99%CIca for Adjusted OR**

Male sex	0.50	0.39-0.64
Learned about HIV/AIDS from print materials	1.59	1.21-2.09
Learned about HIV/AIDS from clinic/health centre	1.87	1.27-2.76
Would tell someone if they were HIV positive	1.82	1.21-2.74
Knows that ARVs can help a person live longer	1.81	1.24-2.63

**Zimbabwe**	**Adjusted OR**	**99%CIca for Adjusted OR**

Male sex	0.70	0.55-0.88
Secondary education or above	1.55	1.20-1.99
Learned about HIV/AIDS from clinic/health centre	1.83	1.28-2.63
Talked often to others about HIV/AIDS	1.57	1.23-1.99

*variables in initial models: age, sex, education level, household income, urban/rural location, food security, condom use with non-regular partner, number of partners in the last year, condom choice-disability, experience of partner violence, feeling at risk for HIV, sources of information about HIV, discussing HIV/AIDS, would tell someone if they knew they were HIV positive, feeling HIV/AIDS is punishment for sinning, thinking those with HIV/AIDS should live apart from others, feeling that telling others you are HIV positive does not help, and knowledge that an HIV infected person live longer when taking ARVs

As shown in Table 4, the two most consistent factors associated with having an HIV test in the last 12 months across the countries were having heard about HIV/AIDS from a clinic or health centre, which was significantly associated with testing in all countries, and having talked often about HIV and AIDS in the last 12 months, which was significantly associated with testing in all countries except South Africa and Zambia.

Also shown in Table 4, other sources of information about HIV and AIDS that were significantly associated with testing uptake in the last year were television (Mozambique and Namibia), print materials (Lesotho, Malawi, Mozambique and Zambia), church (Botswana, Lesotho, Namibia), family members (Lesotho, South Africa and Tanzania) and friends (Botswana). In Mozambique, Tanzania and Zimbabwe, people with more than primary education were significantly more likely to have been tested in the last year than those with less education. In half of the countries (Lesotho, Mozambique, Swaziland, Tanzania and Zambia) those with the knowledge that ART can help a person live longer were significantly more likely to have been tested than those without this knowledge.

### Factors related to intention to test

Among those who had not gone for testing, none of the HIV risk factors we examined (multiple partners, lack of condom use, intimate partner violence and condom use choice disablement) was associated with intention to be tested for HIV, after taking other factors into account. Those who perceived themselves to be at risk of HIV were more likely to intend to be tested (OR 1.6, 99% CIca 1.44-1.83); as were those who knew that ART can help a person live longer (OR 1.67, 99%CIca 1.05-2.66) and those who talked to others about HIV/AIDS (OR 1.24, 99% CIca 1.04-1.47).

## Discussion

People at higher risk of HIV (females, those not always using condoms, those with multiple sexual partners, those who had experienced IPV, and those choice-disabled for condom use) were generally *not *more likely to have been tested in the last year when other factors related to testing were taken into account.

### Gender

Several *s*tudies from Africa [12,20,39] have found that women are more likely to get tested than men. The finding is not uniform. For example, studies conducted in a township of Cape Town, South Africa [22] and rural Uganda [40] did not find that women were more likely to be tested than men. In our study, we found that women were more likely than men to have been tested for HIV in six of the 10 countries. We did not find a significant difference between male and female intention to test among those who had not previously been tested. Some of the explanation for the higher testing rate among women could be their more frequent interaction with health services, including antenatal care and PMTCT programmes [41,42]. However, even when antenatal HIV testing is done under a routine opt-out system, not all women get tested and not all return for their test result or start PMTCT if HIV positive [43].

As women are at higher risk of HIV infection in southern Africa, it is encouraging that they are accessing testing. But perhaps more important to consider than differences in testing rates between men and women are the specific barriers to testing for each sex - such as why men often report waiting until they are symptomatic before choosing to get tested for HIV [41,44] and why some women still do not get tested even as testing is offered to them as part of other care.

### Risky sexual behavior

Several studies have found that people are more likely to be tested if they have risky sexual behaviours, such as having multiple partners [12], sex with a high risk partner [18], or inconsistent use of condoms [39]. Again, this is not consistent across all studies [42,45]. Our study found no significant associations between risky sexual behaviours and testing uptake, or intention to test among those who had not previously been tested. Yet those who have risky sexual behaviours represent a key target group for equity in testing. People who ignore both messages to change high risk behaviours and messages to be tested may be hard-core "risk takers" or they may be choice-disabled and unable to implement protective actions. Either way, new strategies to reach them are needed.

### HIV discussion and stigmatising attitudes

We found in most of the 10 countries that people who had talked to others about HIV were more likely to have been tested in the last year. Other authors have also found the ability to talk about and discuss HIV and AIDS to be associated with HIV testing uptake. Men in Uganda who had discussed HIV prevention with their spouse [14] and youth in South Africa who had talked to their parents about HIV/AIDS [39] were more likely to have been tested. Similarly, frequent conversations about HIV were positively associated with testing in Tanzania, Zimbabwe, South Africa and Thailand [42]. This fits with the behaviour change "cascada" model of intermediate outcomes: **c**onscious knowledge, **a**ttitudes, **s**ubjective norms, intention to **c**hange, **a**gency/self efficacy, **d**iscussion, and **a**ction. This model formed the basis for an intervention to increase uptake of childhood vaccination tested in a randomised cluster controlled trial in a district of Pakistan [46,47]. An important element of the model is Discussion as the immediate precursor of Action. Stimulating discussion was the key element of the RCCT in Pakistan [47,48]. In four districts of Pakistan, parental involvement in discussions about vaccination was related to the likelihood of young children being vaccinated [48] and in India adding interpersonal discussion improved on the KAP (knowledge-attitudes-practice) model for changing HIV risk behaviour [49].

Related to discussion is the issue of stigma, which UNAIDS has identified as a barrier to testing [21]. Studies conducted in South Africa [22] and Botswana [39] found that people holding stigmatising attitudes towards people living with HIV and AIDS were less likely to have been tested for HIV. We did not find an association between stigmatising attitudes and testing in our study; it could be that the two questions we asked about stigma did not reflect the relevant aspects of this complex phenomenon.

### Information from clinics and testing

We found that those who had heard about HIV or AIDS from a clinic or health centre were more likely to have been tested for HIV in the last 12 months. This probably reflects being offered testing while having contact with the health services, perhaps for other reasons (including, for example, antenatal care). This is in line with other studies that report a positive association between health clinic contact and testing uptake [39,41]. The equity challenge remains to increase uptake amongst those at risk of HIV who do *not *have contact with the health care system.

## Limitations

There are several limitations to this study. The cross sectional design only allows us to report associations and limits what we can conclude about causality. Put simply, we cannot tell which came first in associations. For example, the association between HIV information from a clinic and testing may be because people went to a clinic to be tested and were given information about HIV at the time. Some variables related to testing were covered only briefly; for example, stigmatising attitudes were assessed based on only two questions, rather than using a validated scale and this may be one reason why we did not find an association between stigmatising attitudes and being tested for HIV. The 42% non-response rate may have introduced a non-response bias. Most of the non-response was because households had no one, or no one in the relevant age range, present. It is possible those households with someone present had a lower employment rate (and socio-economic status) than those with no one home. Since the study's main conclusions are based on internal associations among the responding households, rather than on absolute frequencies among the responders, they are unlikely to be strongly affected by any non-response bias. It could be argued that multiple significance testing would lead to several significant associations by chance alone. We reduced this possibility by using 99% rather than 95% confidence intervals. Our study did not investigate what might have increased testing among those most at risk of HIV. Studies in Kenya [50], Malawi [51], Uganda [52], and Zambia [53] have shown much higher rates of testing uptake from home-based testing initiatives than from clinic-based testing. Home-based testing may reach people who are not normal users of health services and can reduce socio-economic inequities in testing uptake [54,55] but it is not clear whether it would increase uptake specifically for those at highest risk of HIV. There are cultural differences between the 10 countries. We undertook a separate analysis for each country and in fact found considerable convergence in the variables related to testing between the countries. Cultural differences will be important when designing interventions intended to increase testing among people at higher risk of HIV.

## Conclusion

HIV testing uptake in 10 southern African countries was not higher among those with higher risk of HIV, taking account of other factors related to testing, yet ideally these people *ought *to test more. HIV testing programmes need to encourage those at the highest risk to get tested. Service providers need to recognise that some people are choice-disabled; they are not able to implement preventive actions and may not feel empowered to get themselves tested.

## Competing interests

The authors declare that they have no competing interests.

## Authors' contributions

SM contributed to instrument design, conducted data analysis and drafted the manuscript. AC contributed to instrument design, data analysis, was responsible for overseeing the project in Southern Africa and contributed to the drafting of the manuscript. NA designed the study, developed the methodology, and contributed to the analysis and the drafting of the manuscript. GL assisted with the analysis, developed a cluster adjustment and contributed to the drafting of the manuscript. All authors read and approved the final manuscript.

## Pre-publication history

The pre-publication history for this paper can be accessed here:

http://www.biomedcentral.com/1472-698X/10/23/prepub

## Supplementary Material

Additional file 1 **Questionnaire**. List of questions from the questionnaire used in the analysis. Click here for file
