# Supplementary material for: Equity in HIV testing: evidence from a cross-sectional study in ten Southern African countries
Source: BMC Int Health Hum Rights. 2010 Sep 13;10:23. doi: 10.1186/1472-698X-10-23 (PMC2945979; doi:10.1186/1472-698X-10-23)
Supplement: Additional file 1 — Questionnaire. List of questions from the questionnaire used in the analysis. [file 1472-698X-10-23-S1.PDF]

Questionnaire (list of questions from questionnaire used in the analysis)

Note age and sex of the interviewee
Rural/urban/capital designation
What is the last grade of education you have completed?
What is your total household income per month?
In this household, did you have enough food in the last week?
Would you have sex with your partner if he/she refused to use a condom?
Do you think your husband/ wife/ partner is at risk of getting HIV?
In the last year, have you and your partner had violent arguments where your partner beat, kicked or slapped you?
Did you learn about HIV from... (radio; tv; books, papers or pamphlets; church; clinic or health centre; class in school; family; friends)
In the last year, how often did you talk with anyone about HIV/AIDS?
Do you think you are at risk of getting HIV?
People with HIV or AIDS must live apart from others in the community (strongly agree, agree, no opinion, disagree, strongly disagree, don't know)
HIV/AIDS is punishment for sinning (strongly agree, agree, no opinion, disagree, strongly disagree, don't know)
Telling people you are HIV positive doesn't help anything (strongly agree, agree, no opinion, disagree, strongly disagree, don't know)
If you knew you were HIV positive, who would you tell?
Can an HIV infected person live longer when taking ARVs?
How often do you use condoms when having sex with any other partner (not your regular one)?
How many sexual partners have you had in the last 12 months?
In the last year, have you gone for an HIV test?
Do you plan to have an HIV test yourself?
Do you know where you can get an HIV test?
